# Change in the symptom profile treated as asthma – two cross-sectional studies twenty years apart

**DOI:** 10.1186/s12931-020-1308-3

**Published:** 2020-02-03

**Authors:** Mihkel Pindus, Hans Orru, Rain Jõgi

**Affiliations:** 1 Institute of Family Medicine and Public Health, University of Tartu, Ravila 19, 50411 Tartu, Estonia; 2 Department of Public Health and Clinical Medicine, Occupational and Environmental Medicine, Umeå University, SE–901 87 Umeå, Sweden; 3 Lung Clinic, Tartu University Hospital, Riia 167, 51014 Tartu, Estonia

**Keywords:** Asthma, Respiratory symptoms, Young adults, Prevalence, Trend, Estonia

## Abstract

**Aims:**

The aims of the study were to investigate prevalence trends of respiratory symptoms, asthma and asthma treatment among young adults in Estonia and to estimate changes in symptom profile among subjects who self-report asthma attacks or use asthma medications.

**Methods:**

Two similar questionnaires on respiratory health were sent to subjects in Tartu, Estonia, aged between 20 and 44 years; first in 1993/94, and then in 2014/15. To study the impact of different respiratory symptoms on asthma diagnosis and treatment, the log–binomial regression was used to estimate the association between ‘attack of asthma’ (as a proxy for current asthma) and respiratory symptoms as well as asthma treatment and respiratory symptoms, adjusted for age, sex and smoking history.

**Results:**

Self–reported prevalence of asthma attack, asthma medication use and nasal allergies increased over the twenty years between studies, whereas there was no change in prevalence of asthma–related symptoms, and the prevalence of most respiratory symptoms either decreased, or remained unchanged. For women experiencing asthma attacks, the prevalence of nasal allergies increased and waking with chest tightness decreased. For men using asthma medication, the prevalence of a wheeze without a cold decreased. Women using asthma medication reported decreased prevalence of waking with chest tightness.

**Conclusion:**

Self-reported asthma attacks and asthma medication use has increased in last 20 years, while the prevalence of most respiratory symptoms either decreased or did not change. It is likely that changes in asthma symptom profile have had an impact on the prevalence of asthma and asthma treatment.

## Background

During the second half of the twentieth Century, surveys repeated in the same location and using similar methods generally showed an increased prevalence of asthma and respiratory symptoms over time, both amongst children [1, 2] and adults [3, 4]. However, results of later studies have been less definitive. Some have demonstrated an increase in both the prevalence of self–reported asthma diagnosis and respiratory symptoms suggestive of asthma; whereas others have shown no change in asthma attacks and asthma–like symptoms [5, 6]. Many have shown an increase in diagnosed asthma and asthma treatment, but a smaller increase [7, 8] or even a decrease in respiratory symptoms [9–12]. Studies using objective measurements have shown an increase in medically–diagnosed asthma and respiratory symptoms, but either no change [8, 13] or a decrease [14] in airway hyper–responsiveness.

How much of the apparent increase in asthma prevalence is real, and how much is attributable to the lack of consistent diagnostic criteria for asthma is a question which dates as far back as the first publications of repeated asthma prevalence surveys. Factors which may change over time and lead to biased estimates include: increased awareness of asthma (both amongst doctors and in the general population) [3]; the availability of safe and effective treatment; increased sensitivity to symptoms [1]; and changes in asthma definitions and diagnostic practice [8].

Relatively few studies have described the change in symptom profile over time for subjects with self–reported asthma, and no clear picture has emerged. However, there does appear to be a trend towards milder cases being diagnosed more often now than previously [7, 11]. The current study aimed, firstly, to investigate prevalence trends for respiratory symptoms, asthma diagnosis and asthma treatment among young adults in Tartu, Estonia. Secondly, the study examined long–term changes in the symptom profile of subjects who either self–report asthma attacks or use asthma medications.

## Methods

### Study site and sample

Similar postal questionnaires were sent, 21 years apart, to two random samples of 3000 subjects, aged between 20 and 44 years and drawn from the population of Tartu, Estonia. In 1993/94, the questionnaire was administered within the framework of the European Community Respiratory Health Survey (ECRHS I) [15]. In 2014/15, it was administered as part of the Study of Health Effects in Oil Shale Industry Area (SOHOS). In both surveys two postal reminders and in 2014/15 additional e-mail reminder was sent.

### Questionnaire

The following abbreviations are used to denote affirmative responses to questions (or question combinations) on the questionnaires:

- ‘Breathlessness while wheezing’: affirmative answers to both “Have you had wheezing or whistling in your chest at any time in the last 12 months?” and “Have you been at all breathless when the wheezing noise was present?”
- ‘Wheeze without a cold’: affirmative answers to both “Have you had wheezing or whistling in your chest at any time in the last 12 months?” and “Have you had this wheezing or whistling when you did not have a cold?”
- ‘Woken with chest tightness’: “Have you woken up with a feeling of tightness in your chest at any time in the last 12 months?”
- ‘Woken by attack of breathlessness’: “Have you been woken by an attack of shortness of breath at any time in the last 12 months”?
- ‘Woken by attack of cough’: “Have you been woken by an attack of cough at any time in the last 12 months?”
- ‘Attack of asthma’: “Have you had an attack of asthma in the last 12 months?”
- ‘Current asthma medication’: “Are you currently taking any medicine (including inhalers or tablets) for asthma?”
- ‘Nasal allergies’: “Do you have any nasal allergies including hay fever?”
- ‘Current smokers’: “Do you smoke? (answer yes even if you only smoke a few cigarettes or pipes per week or if you quit smoking for less than one year).”
- ‘Ex–smokers’: “Are you an ex–smoker?”
- ‘Asthma–related symptoms’: affirmative answer to either ‘Breathlessness while wheezing’ or ‘Wheeze without a cold’.

‘Asthma–related disorder’: affirmative answer to either ‘Attack of asthma’ or ‘Current asthma medication’.

### Statistical analysis

The statistical analysis was performed using Stata 12.0 (StataCorp LLC). Both crude and standardised prevalence’s of respiratory symptoms were calculated. Standardised prevalence’s were based on the population age–sex distribution for Tartu in 1993. Differences between two prevalence’s was analysed with two-sample test for proportions and trend analysis for proportions was used to analyse linear trend over more than two proportions. A *p* value <0.05 was considered statistically significant. To study the impact of different respiratory symptoms on asthma diagnosis and treatment log–binomial regression was used to estimate the association between ‘attack of asthma’ (as a proxy for current asthma) and respiratory symptoms as well as asthma treatment and respiratory symptoms, adjusted for age, sex and smoking history.

## Results

The participation rate was significantly higher in 1993/94 (83.0%) than in 2014/15 (35.9%). The response rate was higher for women than for men in both surveys. Mean age in 1993/94 was lower than in 2014/15 (30.2 vs 32.6 years respectively) (Table 1). Mean age of the non-responders in 1993/94 was 29.8 years and 2014/15 32.6 years. All results are based on subjects’ self–reported responses to the questionnaires.

**Table 1:** Characteristics of study sample and respondents by study year and sex

	Women	Men	Total
ECRHS I 1993/94			
Sample	1498	1502	3000
Excluded	19	18	37
Participated	1272	1188	2460
Response rate, %			
Absolute	84.9	79.1	82.0
Adjusted	86.0	80.1	83.0
Mean age of respondents, years	30.3	30.1	30.2
SOHOS 2014/15			
Sample	1498	1502	3000
Excluded	165	119	284
Participated	607	369	976
Response rate, %			
Absolute	40.5	24.6	32.5
Adjusted	45.5	26.7	35.9
Mean age of respondents, years	33.0	32.0	32.6

Excluded = have moved abroad, unable to answer, or died; Absolute Response Rate = Participated/Sample; Adjusted Response Rate = Participated/(Sample–Excluded)

There was significant decrease in current smoking between surveys (45.7% in 1993/94, 20.3% in 2014/15) and increase in non-smokers and ex-smokers. The prevalence of ‘current smokers’ decreased both for women (from 34.2 to 15.3%) and men (from 58.1 to 28.5%) (Table 2).

**Table 2:** Basic characteristics of participants (%) by age group, sex and all subjects

	Year	Age groups, years					*P*-value**	Sex		*P*-value***	All
		20–24	25–29	30–34	35–39	40–44		Women	Men		
Women	1993/94	51.7	49.6	51.3	53.6	53.4	0.372	^a^	^a^	^a^	51.7
	2014/15	55.4	56.4	68.5*	62.6*	65.1*	0.031	^a^	^a^	^a^	62.2*
Non-smokers	1993/94	51.2	41.1	37.6	31.9	45.4	<0.001	54.6	29.2	<0.001	42.3
	2014/15	57.4	49.2*	59.2*	52.9*	59.3*	0.404	63.8*	42.6*	<0.001	55.7*
Ex-smokers	1993/94	7.7	11.0	13.5	15.7	15.6	<0.001	11.2	12.7	0.261	12.0
	2014/15	21.6*	25.4*	24.7*	24.6*	23.0*	0.950	20.9*	29.0*	0.004	24.0*
Current-smokers	1993/94	41.2	47.9	48.9	52.4	38.9	0.347	34.2	58.1	<0.001	45.7
	2014/15	21.0*	25.4*	16.2*	22.5*	17.7*	0.273	15.3*	28.5*	<0.001	20.3*

*p < 0.05 between surveys

**Test for trend by age group

***Difference in prevalence between women and men

^a^Not applicable

The prevalence of ‘attack of asthma’, ‘current asthma medication’ and ‘nasal allergies’ increased between 1993/94 and 2014/15. There was no change in the prevalence of ‘asthma–related symptoms’. The prevalence of most respiratory symptoms either decreased or did not change (Table 3).

**Table 3:** Prevalence (%) of respiratory symptoms, attack of asthma, asthma medication use and smoking by age groups, sex and among all subjects

	Year	Age groups, years					*P*-value**	Sex		*P*-value***	All
		20–24	25–29	30–34	35–39	40–44		Women	Men		
Breathlessness while wheezing	1993/94	7.5	5.9	7.1	8.5	10.7	0.052	7.6	7.8	0.831	7.7
	2014/15	6.9	4.1	6.6	9.7	7.5	0.213	5.9	8.6	0.107	6.9
Wheeze without cold	1993/94	13.1	11.9	11.9	15.0	11.6	0.968	10.1	15.6	<0.001	12.7
	2014/15	11.0	5.6*	7.8	8.7*	11.9	0.317	6.2*	13.3	<0.001	8.9*
Waking with chest tightness	1993/94	11.1	11.6	13.9	14.4	19.8	<0.001	16.4	10.4	<0.001	13.5
	2014/15	12.5	11.9	9.1	16.2	18.1	0.038	14.7	11.3	0.134	13.5
Woken by attack of breathlessness	1993/94	5.6	6.9	8.2	9.7	10.8	0.001	8.8	6.7	0.048	7.8
	2014/15	8.3	9.3	6.5	11.4	13.7	0.052	9.1	11.1*	0.313	9.8
Woken by attack of cough	1993/94	37.7	41.2	45.1	46.6	42.4	0.011	44.2	39.6	0.021	42.0
	2014/15	33.6	37.1	35.2*	34.1*	43.4	0.129	40.1	31.6*	0.008	36.9*
Attack of asthma	1993/94	2.2	1.6	1.1	1.7	2.7	0.873	1.8	1.9	0.927	1.9
	2014/15	4.8	3.6	4.3*	2.7	2.5	0.213	2.8	4.7*	0.127	3.5*
Current asthma medication	1993/94	0.9	0.7	0.4	0.5	0.6	0.414	0.9	0.4	0.180	0.7
	2014/15	4.8*	0.5	3.0*	1.1	2.5	0.395	1.9	3.1*	0.231	2.3*
Nasal allergies	1993/94	16.3	17.6	17.1	20.2	18.3	0.199	17.0	18.4	0.376	17.7
	2014/15	26.5*	21.7	22.2	23.1	27.5*	0.597	25.5*	21.6	0.171	24.0*
Asthma-related symptoms	1993/94	5.3	4.3	4.0	6.1	6.4	0.376	4.6	5.6	0.293	5.1
	2014/15	7.1	3.2	4.2	6.4	6.4	0.600	3.4	8.5	0.001	5.3
Asthma-related disorder	1993/94	2.2	1.8	1.1	2.2	2.7	0.721	2.0	2.0	0.958	2.0
	2014/15	5.5*	3.6	5.2*	2.7	4.0	0.461	3.7*	5.0*	0.339	4.2*

*p < 0.05 between surveys

**Test for trend by age group

***Difference in prevalence between women and men

The prevalence of night symptoms (woken with chest tightness, woken by attack of breathlessness or woken by cough) increased with age in both surveys. There was no significant age trend in any other symptoms. In both surveys wheeze without cold was more prevalent among men while night symptoms were more prevalent in women. Some sex differences in the results were observed between 1993/94 and 2014/15. The prevalence of ‘wheeze without a cold’ decreased in women, whereas the prevalence of ‘woken by attack of breathlessness’ increased in men (Table 3). Age- and sex-standardized prevalence’s are presented in an Additional file 1.

**Fig. 1:**
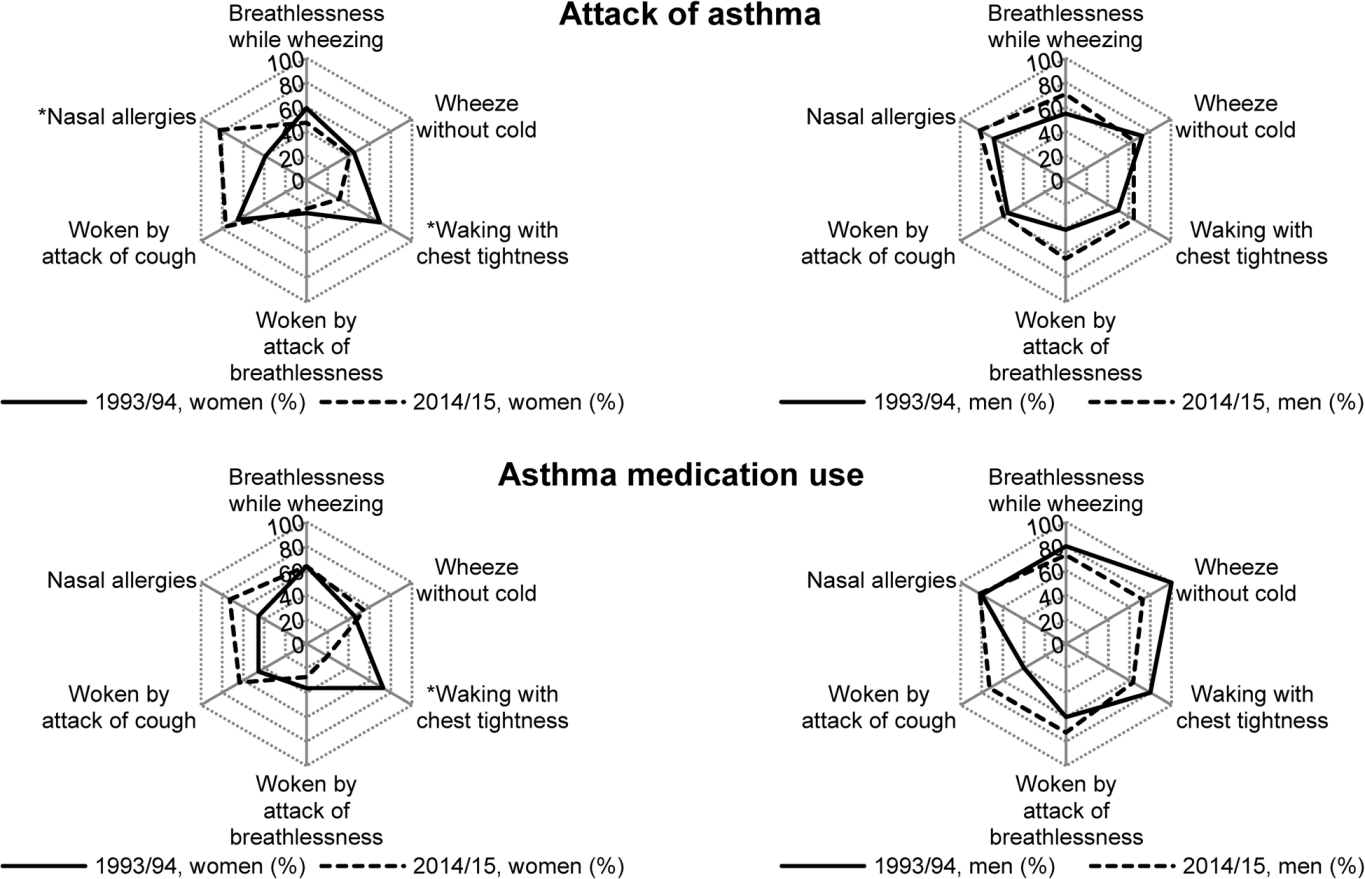
Prevalence (%) of respiratory symptoms in women and men with attack of asthma and asthma medication use. *p < 0.05 from Chi-square test

Changes in the symptom profile among those either reporting ‘attack of asthma’ or who were on ‘current asthma medication’ were also observed between 1993/94 and 2014/15 (see Fig. 1). For women reporting ‘attack of asthma’, the prevalence of ‘nasal allergies’ increased, whereas ‘woken with chest tightness’ decreased. Women on ‘current asthma medication’ reported decrease in ‘woken with chest tightness’. No changes in symptom profile was seen among men.

The results of the regression analysis are shown in Tables 4 and 5. There were significant differences in the associations between respiratory symptoms and self-reported attacks of asthma and asthma treatment between two surveys. In 1993/94 self-reported attack of asthma was associated with wheeze without cold and waking with chest tightness but in 2014/15 with woken by attack of breathlessness. Nasal allergies were more important risk factor for attack of asthma in 2014/15 than in 1993/94. Breathlessness while wheezing was associated with attack of asthma in both surveys. In 1993/94 asthma medication use was positively associated with waking with chest tightness (OR = 5.97, 95% CI 1.56–22.90; ref. no asthma medication users) while in 2014/15 this association had turned to negative (OR = 0.28, 95% CI 0.11–0.69). In 2014/15 but not in 1993/94 asthma medication use was associated with waking by attack breathlessness and waking by attack of cough. Smoking history was negatively associated with asthma medication use in 2014/15.

**Table 4:** Association between self-reported attack of asthma and respiratory symptoms adjusted for age, smoking status and sex

Independent variables	Dependent variables (RR, 95% CI)	
	Attack of asthma 1993/94	Attack of asthma 2014/15
Breathlessness while wheezing	3.42 (1.65–7.07)**	5.02 (1.78–14.17)**
Wheeze without cold	4.05 (2.02–8.10)***	1.40 (0.57–3.45)
Waking with chest tightness	3.36 (1.65–6.83)**	0.84 (0.39–1.80)
Woken by attack of breathlessness	0.92 (0.47–1.80)	2.22 (1.06–4.64)*
Woken by attack of cough	1.08 (0.61–1.90)	1.75 (0.98–3.14)
Nasal allergies	2.80 (1.57–4.98)***	6.68 (2.67–16.67)***
Age	0.98 (0.95–1.02)	0.97 (0.94–1.00)
Smoking history	0.77 (0.56–1.07)	1.01 (0.72–1.40)

All independent variables were added into the models all at once. Relative risk (RR) with 95% Confidence Intervals (CI). *p < 0.05, **p < 0.01, ***p < 0.001

**Table 5:** Association between self-reported use of asthma medication and respiratory symptoms, adjusted for age, smoking status and sex

Independent variables	Dependent variables (RR, 95% CI)	
	Asthma medication use 1993/94	Asthma medication use 2014/15
Breathlessness while wheezing	6.29 (1.67–23.70)**	9.35 (2.19–40.03)**
Wheeze without cold	3.18 (0.97–10.36)	3.66 (1.01–13.26)*
Waking with chest tightness	5.97 (1.56–22.90)**	0.28 (0.11–0.69)**
Woken by attack of breathlessness	1.13 (0.40–3.18)	3.57 (1.33–9.56)*
Woken by attack of cough	0.49 (0.19–1.26)	2.29 (1.24–4.23)**
Nasal allergies	2.65 (1.05–6.69)*	3.86 (1.40–10.68)**
Age	0.95 (0.89–1.02)	0.94 (0.90–0.98)**
Smoking history	0.67 (0.40–1-13)	0.52 (0.37–0.74)***

All independent variables were added into the models all at once. Relative risk (RR) with 95% Confidence Intervals (CI). *p < 0.05, **p < 0.01, ***p < 0.001

### Sensitivity analysis

We compared the prevalence of respiratory symptoms; attacks of asthma and asthma medication use among early (<4 weeks) and late (>8 weeks) respondents. We did not find any difference except current asthma medication that was less prevalent among late responders in 1993/94 (Additional file 1).

To estimate the influence of the change in smoking prevalence between surveys, we compared the prevalence of respiratory symptoms among lifelong non-smokers. The prevalence of respiratory symptoms was generally lower among lifelong non-smokers in both surveys. The general pattern of the changes in the prevalence of respiratory symptoms was fairly similar compared to the prevalence in whole study population. The results are presented in Additional file 1.

## Discussion

The main results of the study were that the prevalence of self-reported asthma attacks, and the use of asthma medication both increased between 1993/94 and 2014/15, whilst the prevalence of most respiratory symptoms either decreased or did not change. This aligns with results from previous similar studies, where increases in asthma attacks and medication use corresponded with a decrease in asthma–related respiratory symptoms [7–11].

It is likely that multiple factors contribute to the increased prevalence of asthma and asthma treatment, such as increased awareness of asthma, and increased diagnostic activity (9). In addition to real increase [1] and better recognition of the condition [2, 8], the ability to perceive and report bronchoconstriction has also changed over time. For example, between 1993 and 1999, Barraclough et al [14] observed no difference in perception of a more than 20% decrease in FEV1 in methacholine testing, whilst the perception of a less than 20% decrease nearly tripled. Moreover, women and subjects with atopic sensitisation had a greater risk of increased sensitivity to the perception of a decrease in FEV1.

Results from previous studies indicate that the symptom profile treated as asthma has become milder over time. In a study by Ekerljung et al [7] among subjects with current asthma, 41% had only one or two indicators (i.e. either symptoms or asthma treatment) of asthma in 2007, as compared with 27% 10 years earlier. A significantly higher proportion of subjects with few symptoms were using asthma medication in 2007, than in 1996. In a repeated prevalence study on children by Anderson et al [16], persistent wheezing increased, whilst severe attacks and chronic disability fell by about half.

The asthma symptom profile also appears to have widened over time. One example to this is “cough variant asthma”. Despite launched already in early 80s [17], it was not mentioned in the first international consensus report on diagnosis and treatment of asthma in 1992 [18]. In GINA 2002 cough variant asthma is addressed in Chapter 5 under paragraph “Particularly difficult diagnostic groups”, while in GINA 2006 cough variant asthma is described already in Chapter 2 under paragraph “Clinical diagnosis”. This widening of the definition of asthma is likely to have affected the prevalence of medically-diagnosed asthma, since cough is a frequent reason for general practitioners’ referral and is often difficult to treat [19].

In the current study, a significant increase in nasal allergies and decrease in chest tightness was observed in the asthma symptom profile for women (Fig. 1). There were significant differences in the associations between respiratory symptoms and self-reported attacks of asthma and asthma treatment between two surveys. The prevalence of attack of cough decreased between surveys, its impact on asthma treatment, however increased.

One of the main risk factors for respiratory symptoms is smoking, the prevalence of which has decreased in many countries over the last few decades [4, 9, 10, 20]. This further complicates the measurement of asthma prevalence, as it is difficult to estimate the extent to which any decrease in respiratory symptoms caused by a decrease in exposure to smoking is counterbalanced by an increase in symptoms caused by the possible increased prevalence of asthma. However, as we did not see differences in the general pattern of the changes in symptom prevalence comparing all responders and lifelong non-smokers (Table 3 and Additional file 1), it is unlikely that described change in the pattern of the prevalence of respiratory symptoms is due to decreased prevalence of cigarette smoking.

Whether the symptom profile of people with self-reported asthma has changed over time, is not well understood. A study by Brogger et al [4] showed consistent results over time, with approximately 70% of subjects with self-reported asthma reporting wheezing, and 60% reporting attacks of breathlessness, both in 1972, and again in 1998–1999. Also, we do not have information on symptoms presence when asthma was diagnosed in our study subjects. We cannot therefore confirm, if the change in asthma symptom profile that we see, is the result in the change in asthma labelling.

Some studies have shown greater increase in asthma prevalence for women than for men [4]. In the current analysis, women with self-reported asthma attacks had a different symptom profile than in men, in both surveys. Men with asthma became more symptomatic overall, whereas the women’s symptom profile changed (e.g. increased nasal allergies and decreased waking with chest tightness). For women, waking with chest tightness, became relatively less important factor for asthma treatment (Fig. 1).

Asthma treatment has changed considerably in last 20 years. Twenty years ago long-acting beta2- adrenomimetics were not on the market, not to mention combination inhalers. As we did not collect detailed information on asthma medication consumption in our surveys, we could not analyse the potential effect of asthma treatment to respiratory symptoms. One could expect that drug availability and bigger choice of effective treatment options would lead to better treatment effect and less symptoms. For example, it would be tempting to explain the reversal of the association between waking with chest tightness and asthma medication use from positive in 1993/94 to negative in 2014/15 with better treatment effect. The general pattern of the change in symptom profile does not, however, seem to support this notion as waking by attack of breathlessness and waking by attack of cough were associated with asthma medication use in 2014/15 but not in 1993/94. It seems unlikely that asthma treatment could considerably decrease some of the respiratory symptoms, while increasing others.

The main limitation of the study is the different participation rates in the first and in the second study and compared to some of the earlier studies [6, 12, 21], the drop in the participation rate in the current study is larger. Participation was especially low among younger men, similarly with other recent studies [12]. Due to low participation rate, some of the substantial changes, e.g. decrease in smoking prevalence, could at least partly be a result of decreasing participation rates. However, we did not see any difference between early and late responders in 2014/15. The main strength of the current study is that similar methods were used with identical validated questions within the same age-span in the same area (city of Tartu) using random weighted sample relevant to actual age distribution among 20–44-year-old adults approximately 20 years apart.

## Conclusion

Self-reported asthma attacks and asthma medication use has increased in last 20 years, while the prevalence of most respiratory symptoms either decreased or did not change. It is likely that changes in asthma symptom profile have had an impact on the prevalence of asthma and asthma treatment. The clinical implications of the findings are to remind that objective measurements of lung function are important if we want to keep asthma definition unchanged in everyday practice and avoid treating every respiratory symptom as “asthma” and that availability of effective asthma treatment can lead to higher prevalence of diagnosed asthma without lowering the prevalence of respiratory symptoms in the population.

## Supplementary information

**Additional file 1: Table S1.** Crude and age- and sex-standardised prevalence’s (%) of respiratory symptoms in ECRHS I and SOHOS study using 1993-year annual population data from Tartu, Estonia. **Table S2.** Difference in prevalence (%) of respiratory symptoms for early (<4 weeks) and late (>8 weeks) questionnaire respondents. **Table S3.** Prevalence (%) of respiratory symptoms, attack of asthma and asthma medication use in women, men among non-smokers. *p < 0.05 from Chi-square test.

## Data Availability

Additional statistical analysis and figures are included as supplementary information files.

## Abbreviations

ECRHS: European Community Respiratory Health Survey
FEV_1_: Forced expiratory volume
SOHOS: Study of Health Effects in Oil Shale Industry Area
