# Supplementary material for: Change in the symptom profile treated as asthma – two cross-sectional studies twenty years apart
Source: Respir Res. 2020 Feb 3;21:41. doi: 10.1186/s12931-020-1308-3 (PMC6998351; doi:10.1186/s12931-020-1308-3)
Supplement: Supplementary file 1 — Additional file 1: Table S1. Crude and age- and sex-standardised prevalence’s (%) of respiratory symptoms in ECRHS I and SOHOS study using 1993-year annual population data from Tartu, Estonia. Table S2. Difference in prevalence (%) of respiratory symptoms for early (<4 weeks) and late (>8 weeks) questionnaire respondents. Table S3. Prevalence (%) of respiratory symptoms, attack of asthma and asthma medication use in women, men among non-smokers. *p < 0.05 from Chi-square test. [file 12931_2020_1308_MOESM1_ESM.pdf]

ONLINE SUPPLEMENTARY MATERIAL

Change in the symptom profile treated as asthma – two cross-sectional studies twenty years apart

Mihkel Pindus, Hans Orru, Rain Jõgi

ONLINE SUPPLEMENTARY TABLE S1 Crude and age- and sex-standardised prevalence's (%) of respiratory symptoms in ECRHS I and SOHOS study using 1993-year annual population data from Tartu, Estonia.

	ECRHS I 1993/94		SOHOS 2014/15	
	Crude	Age- & sex-standardised	Crude	Age- & sex-standardised
Breathlessness while wheezing	7.7	7.7	6.9	7.2
Wheeze without cold	12.7	12.7	8.9	9.0
Waking with chest tightness	13.5	13.7	13.5	12.7
Woken by attack of breathlessness	7.8	7.9	9.8	9.2
Woken by attack of cough	42.0	42.1	36.9	34.7
Attack of asthma	1.9	1.8	3.5	3.9
Current asthma medication	0.7	0.6	2.3	2.4
Nasal allergies	17.7	17.8	24.0	23.5
Current smokers	45.7	45.9	20.3	23.7
Ex-smokers	12.0	12.0	24.0	20.9
Asthma-related symptoms	5.1	5.1	5.3	5.5
Asthma-related disorder	2.0	1.9	4.2	4.5

Sensitivity analysis

ONLINE SUPPLEMENTARY TABLE S2 Difference in prevalence (%) of respiratory symptoms for early (<4 weeks) and late (>8 weeks) questionnaire respondents.

	ECRHS I			SOHOS		
	< 4 weeks	>8 weeks	*P*-value	< 4 weeks	>8 weeks	*P*-value
Breathlessness while wheezing	7.7	6.2	0.229	7.4	6.6	0.729
Wheeze without cold	13.4	12.9	0.794	8.6	11.0	0.328
Waking with chest tightness	12.7	13.3	0.726	11.3	14.3	0.284
Woken by attack of breathlessness	7.6	9.2	0.221	10.9	12.8	0.488
Woken by attack of cough	40.5	40.5	0.993	34.0	40.0	0.136
Attack of asthma	1.4	1.4	0.929	4.7	3.1	0.333
Current asthma medication	0.8	0.1	<0.05	3.5	2.2	0.351
Nasal allergies	17.8	16.9	0.630	22.3	25.5	0.371

ONLINE SUPPLEMENTARY TABLE S3 Prevalence (%) of respiratory symptoms, attack of asthma and asthma medication use in women, men among non-smokers. *p<0.05 from Chi-square test.

	Sex	Year	
		Women	Men
Breathlessness while wheezing	1993/94	6.9	3.5
	2014/15	4.7	3.9
Wheeze without cold	1993/94	6.3	5.3
	2014/15	2.6*	5.8
Waking with chest tightness	1993/94	16.1	8.4
	2014/15	10.3*	9.6
Woken by attack of breathlessness	1993/94	8.7	4.1
	2014/15	6.3	9.6*
Woken by attack of cough	1993/94	37.5	28.3
	2014/15	34.4	26.9
Attack of asthma	1993/94	2.0	1.7
	2014/15	1.8	3.8
Current asthma medication	1993/94	1.3	0.0
	2014/15	2.1	3.2
Nasal allergies	1993/94	17.2	17.9
	2014/15	25.9*	20.7
Asthma-related symptoms	1993/94	3.3	2.2
	2014/15	1.6	3.9
Asthma-related disorder	1993/94	2.2	1.7
	2014/15	2.7	4.5

*p< 0.05 between surveys.
